# Entropies in Electric Circuits

**DOI:** 10.3390/e27010073

**Published:** 2025-01-15

**Authors:** Angel Cuadras, Victoria J. Ovejas, Herminio Martínez-García

**Affiliations:** Electronics Engineering Department (DEEL), Energy, Power and Integrated Circuits (EPIC), Escola d’Enginyeria de Barcelona Est (EEBE), Universitat Politècnica de Catalunya—BarcelonaTech (UPC), Av. d’Eduard Maristany, 16 Edifici A Campus Besòs, 08029 Barcelona, Spain; victoria.julia.ovejas@upc.edu (V.J.O.); herminio.martinez@upc.edu (H.M.-G.)

**Keywords:** configurational entropy, thermodynamic entropy, constructal law, electric circuit, parallel and series equivalents, maximum power transfer theorem

## Abstract

The present study examines the relationship between thermal and configurational entropy in two resistors in parallel and in series. The objective is to introduce entropy in electric circuit analysis by considering the impact of system geometry on energy conversion in the circuit. Thermal entropy is derived from thermodynamics, whereas configurational entropy is derived from network modelling. It is observed that the relationship between thermal entropy and configurational entropy varies depending on the configuration of the resistors. In parallel resistors, thermal entropy decreases with configurational entropy, while in series resistors, the opposite is true. The implications of the maximum power transfer theorem and constructal law are discussed. The entropy generation for resistors at different temperatures was evaluated, and it was found that the consideration of resistor configurational entropy change was necessary for consistency. Furthermore, for the sake of generalization, a similar behaviour was observed in time-dependent circuits, either for resistor–capacitor circuits or circuits involving degradation.

## 1. Introduction

Energy is inextricably linked to entropy, as its transformations are governed by the second law of thermodynamics. Although this statement applies to all forms of energy, the application of entropy is generally found in thermal energy conversion [1]. Its use in electrical circuit analysis is much less common [2] and a general agreement on its understanding has yet to be established. For instance, it is claimed that either entropy generation is maximal [3,4] or minimal [5,6]. Moreover, the state of the art on entropy in electrical circuits also describes the organization of the circuit network configuration in terms of entropy, i.e., how the electrical elements are interrelated.

Considering that the theory of circuit analysis is well established, based on the conservation of charge and energy, and ultimately described by Kirchhoff’s circuit laws, we aim to evaluate if the use of entropy can clarify the intrinsic behaviour of circuit analysis in cases where the circuit can be at different temperatures, such as in the case of a battery. Thus, we will briefly describe the most valuable contributions of entropy from both a network point of view and thermodynamic point of view, in order to relate them in this contribution, taking into account that the total entropy of the system will be the sum of all forms of entropy [7], and always keeping in mind that, as Jaynes explicitly stated, “We must warn at the outset that the major occupational disease of this field is a persistent failure to distinguish between the information entropy, which is a property of any probability distribution, and the experimental entropy of thermodynamics, which is instead a property of a thermodynamic state, […] But in case the problem happens to be one of thermodynamics, there is a relationship between them” [8].

### 1.1. Network Entropy

Electrical networks have been investigated in terms of the drunkard’s walk problem, a well-studied situation in the theory of probability [9,10,11,12], so that resistor networks can be interpreted in terms of probability. Nachmias developed a mathematical description of the physical network laws from voltage and current harmonic functions [12]. Perelson described thermodynamics in terms of networks and Kirchhoff’s law [13], which is similar to the approach of this contribution, in which we apply thermodynamics to networks. Since a network is a connected graph endowed with positive edges, such as resistances, there is an extensive body of literature on entropy in graphs theory [14,15], with different entropy-based measures based on network invariants, such as the number of vertices or the vertex degree sequence. Moreover, Anand and Bianconi studied entropy in networks [16], comparing Gibb’s, Shannon and Von Neumann entropy in networks describing microcanonical and canonical examples. Their results have been applied in several fields, such as robotics [17], biophysics [18], and stock markets [19]. Finally, though several references can be found for resistor networks, the presence of capacitors or inductors is much less common, being limited to the modelling of power lines [20], DC–DC converters [21], or with quantum effects [22], without playing an active role in the description of network entropy.

### 1.2. Thermal Entropy in One Resistor

In the framework of irreversible thermodynamics, the entropy associated with energy conversion (thermodynamic entropy) in a resistor *R* is described by Ohm’s law, *V* = *I R*, that is, by the relationship between current *I* (flow) and voltage *V* (gradient). It is well established that, when the current flows through the resistor, electrical energy is converted to heat and thermodynamic entropy *S*_therm_ is generated in the resistor at a rate of

(1) $${\dot{S}}_{therm}=\frac{dS_{therm}}{dt}=\frac{IV}{T_{R}}=\frac{P_{diss}}{T_{R}}$$

where *T*_R_ is the resistor temperature [23,24] and *P*_diss_ is the dissipated power. In this basic configuration, we have only one degree of freedom and *S*_therm_ describes how electrical energy is converted into thermal energy. It is worth noting that the entropy that reaches the environment at a temperature *T* (cold source) is given by Equation (1) plus the entropy generated from transferring the heat from the resistor (hot source) to the environment:

(2) $${\dot{S}}_{therm\_env}={\dot{S}}_{therm}+P_{diss}\left(\frac{1}{T}-\frac{1}{T_{R}}\right)=\frac{P_{diss}}{T}$$

which is simply the Gouy–Stodola theorem [25]. In terms of entropy balance, the entropy change at the resistor $∆{\dot{S}}_{R}$, is due to entropy generation ∆_i_*S* and exchanged entropy with the environment ∆_e_*S* [23]:

(3) $$∆{\dot{S}}_{R}={∆}_{i}\dot{S}+{∆}_{e}\dot{S}$$

The resistor *R* where $∆{\dot{S}}_{R}$ is generated must be understood as the system in which the energy conversion from electrical to thermal takes place, as here, no physical change in the resistor structure is considered.

### 1.3. Literature Review of Thermal Entropy in Two Resistors

**Figure 1:**
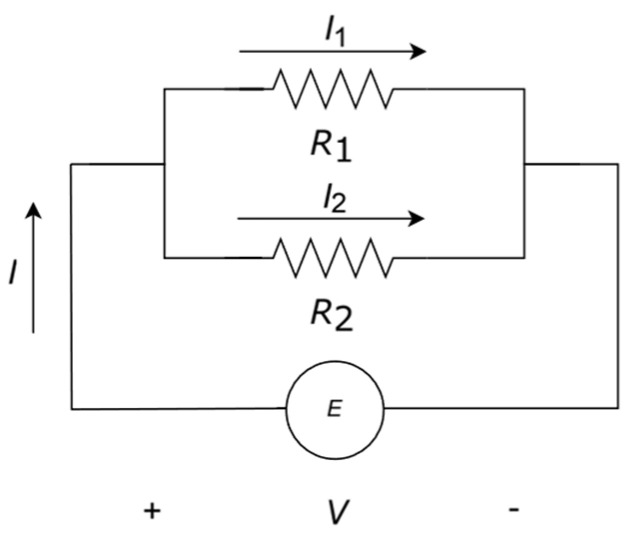
Current divider with two resistors in parallel. *E* describes a power source, either of voltage or current.

When two resistors are connected in parallel to a power source (Figure 1) a current divider is created.

The following derivation to find the electronic behaviour of the current divider from the minimisation of the entropy generation rate can be found in the literature [26,27]. The dissipated power *P*_diss_ is given by the following:

(4) $$P_{diss}=I_{1}V+I_{2}V$$

where *V* is the voltage drop across the two resistors. The parallel configuration introduces the following constraint:

(5) $$I=I_{1}+I_{2}$$

For linear relationship between fluxes and gradients (Ohm’s law):

(6) $$V=I_{1}R_{1}\;and\;V=I_{2}R_{2}$$

And thus, the entropy generation rate is written as follows:

(7) $${\dot{S}}_{therm}=\frac{I_{1}V+I_{2}V}{T},$$

where *T* is assumed to be constant in an isothermal process with no temperature change at the resistors. For a steady state, it is claimed that, if the entropy generation rate is minimized and equal to zero

(8) $$\frac{d{\dot{S}}_{therm}}{dI_{1}}=\frac{1}{T}\frac{dP}{dI_{1}}=\frac{1}{T}\frac{d}{dI_{1}}\left(I_{1}V+I_{2}V\right)=\frac{1}{T}\frac{d}{dI_{1}}\left(I_{1}^{2}R_{1}+{I_{2}^{2}R}_{2}\right)=\;\frac{1}{T}\frac{d}{dI_{1}}\left(I_{1}^{2}R_{1}+{(I-I_{1}{)}^{2}R}_{2}\right)=\frac{1}{T}\left(2I_{1}R_{1}+{2I}_{1}R_{2}-2IR_{2}\right)=0$$

Hence:

(9) $$\left(I_{1}R_{1}+I_{1}R_{2}-IR_{2}\right)=0$$

it leads to the expression of the current divider

(10) $$I_{1}=\frac{R_{2}}{R_{1}+R_{2}}I$$

Another approach to entropy in parallel resistor circuits is based on variational analysis. A review shows that maximum entropy production (MaxEP) and minimum entropy production principles (MinEP) can be applied [28] as long as the temperature is homogeneous [29]. Furthermore, Christen [27] pointed out the superiority of MaxEP as it is applicable far from the equilibrium, while MinEP is restricted to near equilibrium, although no direct application to the electrical network is presented for MaxEP. Yet, a dynamic approach based on the theorem of minimum entropy production in linear systems with inertial effects showed that entropy can be split into excess entropy, related to dissipation and total entropy, including the inertial effects [30].

### 1.4. Degradation

Entropy is also present in systems undergoing degradation. Degradation is the process in which the input energy in a system modifies the internal structure of the system. Basaran pioneered its introduction in soldered joints [31,32] and developed the unified mechanics theory as an attempt to combine mechanics and thermodynamics into a single discipline. Naderi et al. [33] applied the theory to mechanical systems and, extending the application from mechanical systems to electrical systems, our group has focused on electrical systems, such as resistors, capacitors, LEDs, and batteries, along with energy degradation in terms of energy efficiency [34,35,36,37,38]. These studies only consider *S*_therm_, and describe degradation to failure with an increase of *S*_therm_ up to a threshold [39].

### 1.5. Literature Gap and Paper Structure

When we reviewed the literature, some concerns were raised about the derivation of Equations (8)–(10) because the temperature is assumed to be the same in both resistors and, therefore, has no effect on the derivation. For the sake of illustration, we consider the counter example where two resistors *R*_1_ and *R*_2_ are at different temperatures, *T*_1_ and *T*_2_. The entropy generation rate at *R*_1_ and *R*_2_, respectively, is as follows:

(11) $${\dot{S}}_{1}=\frac{I_{1}V}{T_{1}}\;and\;{\dot{S}}_{2}=\frac{I_{2}V}{T_{2}}$$

Then, the total entropy generation rate is as follows:

(12) $${\dot{S}}_{therm}={\dot{S}}_{1}+{\dot{S}}_{2}=\frac{I_{1}V}{T_{1}}+\frac{I_{2}V}{T_{2}}$$

and minimising the entropy generation rate:

(13) $$\frac{d\dot{S}}{dI_{1}}=0=\frac{d}{dI_{1}}\left(\frac{I_{1}V}{T_{1}}+\frac{I_{2}V}{T_{2}}\right)=\frac{d}{dI_{1}}\left(\frac{I_{1}^{2}R_{1}}{T_{1}}+\frac{{(I-I_{1}{)}^{2}R}_{2}}{T_{2}}\right)=\frac{2I_{1}R_{1}}{T_{1}}+\frac{{2I}_{1}R_{2}-2IR_{2}}{T_{2}}$$

Hence,

(14) $$I_{1}=I\frac{\frac{R_{2}}{T_{2}}}{\left(\frac{R_{1}}{T_{1}}+\frac{R_{2}}{T_{2}}\right)}$$

The current depends on the temperature of each resistor. Therefore, only in the case that *T*_1_= *T*_2_ we recover the expression obtained from Kirchhoff’s law. The discrepancy between Equation (14) and Kirchhoff’s law was tested experimentally with two variable resistors in order to have the same resistance at different temperatures (i.e., 1 kΩ at 25 °C and 50 °C), finding that the current profile obeys Kirchhoff’s current law in Equation (10) and minimum energy dissipation [40] but not the entropy minimisation described by Equation (14).

Thus, it seems clear from the literature that a deeper analysis of the entropy of electrical circuits is worthy of further investigation for a number of reasons. Firstly, electrical circuits are ubiquitous in modern technology. Secondly, constant temperature is a strong constraint that could have powerful applications in, for example, battery management, where the role of entropy is ignored. Finally, the methodology can be straightforwardly generalised to other linear systems, as equivalent electrical circuits can be found in thermal management and biological applications [13], among other applications. Within this framework, we aim to fill the gap of the application of the fundamental second law of thermodynamics in electrical circuits. For this, we need to identify the behaviour of the circuit in terms of entropy. Thus, the objective of this manuscript is to evaluate the entropy of simple electronic circuits in order to elucidate the relevance of two different types of entropy: the network configuration expressed in terms of *S*_config_, and the energy conversion expressed in terms of *S*_therm_. The innovation of the manuscript is, thus, to simultaneously investigate the entropy related to circuit design with the entropy related to energy transformation in the same circuit and find their correlations in different types of circuits. It will also be possible to infer the impact of circuits in non-equilibrium, that is, that different elements of the circuit are at different temperatures.

The paper is organised as follows. First, we explain the methodology on how to calculate network entropy for circuits with resistors and capacitors, then we present the results of entropy correlation for parallel and series configurations with voltage and current sources. We continue with two tree-shaped networks and two source circuits to clarify the influence of element disposition in thermal dissipation. Finally, we evaluate the impact of network configuration due to time dependent circuits in *R-C* configurations and degradation.

## 2. Materials and Methods

As mentioned in the introduction, we are concerned with two types of entropy, *S*_config_, related to network configuration, and *S*_therm_, related to energy conversion. *S*_therm_ is computed as described above in Equation (1) in terms of voltage, current, and temperature. As we only focus steady state circuits, we obtain that $S_{therm}={\dot{S}}_{therm}\;\Delta t$, and unless stated different, we take Δ*t* = 1 s.

*S*_config_ is the new term we propose to characterize the circuit. We calculated it in terms of the probability that an element of the network configurations takes place in the analysis, which is described by Kirchhoff’s and Ohm’s law. We describe the analysis of the entropy calculation for two resistors in parallel, in the configuration shown in Figure 1. Applying Kirchhoff’s and Ohm’s laws, we can write the relationship between currents and voltages in terms of a network matrix:

(15) $$\left[\begin{array}{c} V\\ V \end{array}\right]=\left[\begin{array}{c} R_{1}\\ 0 \end{array}\begin{array}{c} 0\\ R_{2} \end{array}\right]\left[\begin{array}{c} I_{1}\\ I_{2} \end{array}\right]$$

The probabilistic interpretation of this expression in terms of conductance can be found in [9] but, for our purposes in relating it to thermal entropy, we retain the resistive form. We aim to calculate the entropy of this matrix. As the matrix is diagonal, we can use the Shannon entropy using the expression (see [41]):

(16) $$S_{config}=-\sum_{i=1}^{n}p\left(x\right)\ln\left(p\left(x\right)\right)$$

Equation (16) thus provides information about the configuration of the system, which in this case has two degrees of freedom. Landauer proposed this approach, but he did not develop it [40]. In this expression, we consider that *p*(*x*) represents the probability of current flowing through one resistor. This is a slightly different interpretation of standard network entropy found in graph theory, which defines the probability of moving between nodes, whereas we define the probability in terms of energy distribution among the different network elements using Ohm’s law. Considering the two resistors of a current divider together, and assuming a constant temperature *T*, the result is as follows:

(17) $$S_{config}=-\left[\frac{R_{2}}{R_{1}+R_{2}}ln\left(\frac{R_{2}}{R_{1}+R_{2}}\right)+\frac{R_{1}}{R_{1}+R_{2}}ln\left(\frac{R_{1}}{R_{1}+R_{2}}\right)\right]$$

It can be seen that the probability of current flowing through one resistor or the other is simply given by the current divider solution given in Equation (10). We consider this expression to describe the configurational entropy of a circuit.

**Figure 2:**
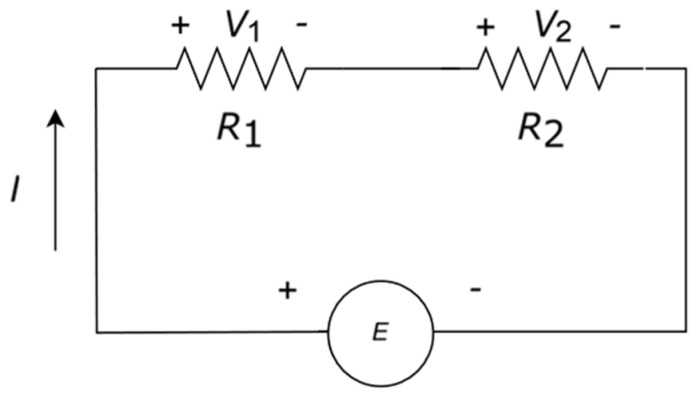
Voltage divider with two resistors in series. *E* stands either for a voltage or current source.

Another typical resistor configuration is two resistors in series (see Figure 2). In this case, the circuit considered is a voltage divider instead of a current divider, defined by the following:

(18) $$V_{1}=\frac{R_{1}V}{R_{1}+R_{2}},\;V_{2}=\frac{R_{2}V}{R_{1}+R_{2}}$$

We want to determine *S*_config_ using the same procedure as that for parallel resistors. The matrix is again diagonal:

(19) $$\left[\begin{array}{c} I\\ I \end{array}\right]=\left[\begin{array}{c} R_{1}\\ 0 \end{array}\begin{array}{c} 0\\ R_{2} \end{array}\right]\left[\begin{array}{c} V_{1}\\ V_{2} \end{array}\right]$$

Therefore,

(20) $$S_{config}=-\left[\frac{R_{1}}{R_{1}+R_{2}}ln\left(\frac{R_{1}}{R_{1}+R_{2}}\right)+\frac{R_{2}}{R_{1}+R_{2}}ln\left(\frac{R_{2}}{R_{1}+R_{2}}\right)\right]$$

**Figure 3:**
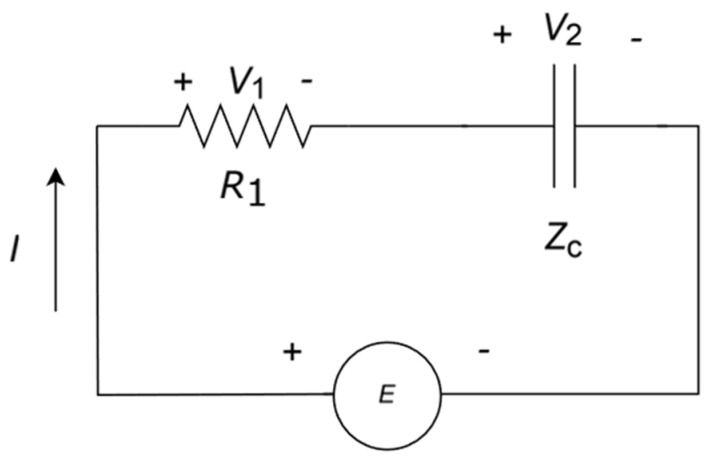
Equivalent series resistance and capacitor with a power source *E*.

In this particular case, *S*_config_ is the same for both parallel and series resistors. We extend this analysis to *R-C* networks, which are less common. Following our considerations on *S*_config_, we notice that this circuit (Figure 3) has only one degree of freedom in the DC analysis and the entropy is zero.

If *E* is time-dependent, e.g., a sinusoidal source in steady state, then the capacitor impedance is frequency-dependent Zc=1iωC, which introduces a new degree of freedom. Accordingly, we can write *S*_config_ as follows:

(21) $$S_{config}=\left|\frac{R_{1}}{R_{1}+Z_{c}}ln\left(\frac{R_{1}}{R_{1}+Z_{c}}\right)+\frac{Z_{c}}{R_{1}+Z_{c}}ln\left(\frac{Z_{c}}{R_{1}+Z_{c}}\right)\right|$$

where ln (*r* e^iθ^) = ln *r* + i*θ* is the principal value of the logarithm. The use of complex Shannon entropy has already been studied [42,43].

To obtain *S*_therm_ for these circuits, we follow the description of the state of the art described in Equation (11). For capacitive networks, *S*_therm_ can be evaluated either in the time domain or in the frequency domain, taking advantage of the invariance of energy and entropy under time transformations. For simplicity, we consider a single sinusoidal excitation signal characterised by its root mean square voltage (*V*_rms_).

For completeness, we also investigate the time dependence in systems undergoing degradation. In this case, we consider that the input electrical energy modifies one of the resistors, *R*_2_, due to either material fatigue or material breakdown. Thus, *S*_config_ is a function of time, and *S*_therm_ is obtained from the time integration of the input electrical power. We consider three different time-dependent functions for *R*_2_ degradation, described by: (i) a linear function,

(22) $$R_{2deg1}\left(t\right)=R_{2}\;t\;Heaviside\left(10-t\right)$$

(ii) a quadratic function:

(23) $$R_{2deg2}\left(t\right)={0.1\;R}_{2}\;t^{2}\;Heaviside\left(10-t\right)$$

or (iii) an exponential function:

(24) $$R_{2deg3}\left(t\right)=0.001\;R_{2}\;e^{t}\;Heaviside\left(10-t\right)$$

which are commonly used in fatigue and breakdown studies. Each function describes material fatigue as a linear, quadratic, or exponential increase in *S*_config_. In addition, we also introduce an entropy threshold discontinuity with a Heaviside function, which describes structure breakdown, in cases that it is reached. The multiplying constants in *R*_2deg2_ and *R*_2deg3_ are chosen to keep the degradation in the same range for *t* = 10 s. We substitute *R*_2_ for *R*_2deg_ in entropy calculations in order to introduce time-dependent degradation in the resistor.

## 3. Results

We present the results for resistor networks, evaluating the relationship of *S*_config_ and *S*_therm_ and relating both types of entropy. We conclude the analysis with two types of time-dependent circuits, *R-C* circuits and systems with degradation.

### 3.1. Entropy in Two Parallel Resistors

**Figure 4:**
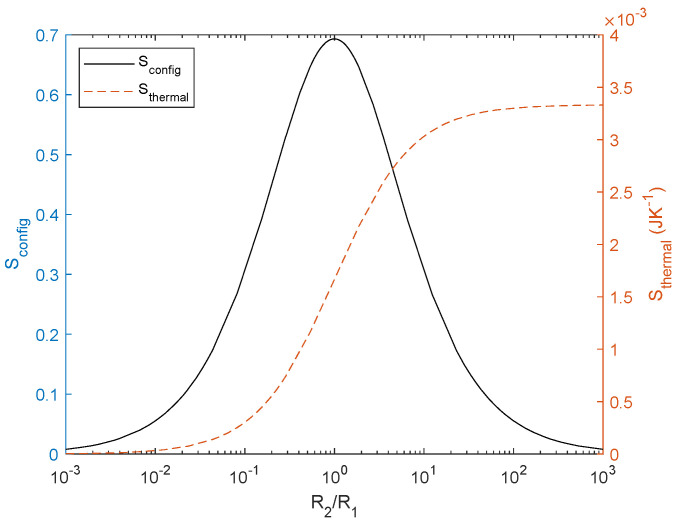
Configurational entropy (left) and thermal entropy (right) as a function of the normalized resistor ratio for a current source of 1 A and integration time of 1 s. *S*_config_ maximum is 0.68 for equal resistors and evolve to 0 when the difference between resistors increases. *S*_therm_ increases with *R*_2_.

We evaluate *S*_config_ for different ratios of resistor pairs (Figure 4) using Equation (17); the maximum entropy is found when the resistors are equal and decreases as their difference increases. Note that the graph is symmetrical with respect to the maximum found for *R*_2_/*R*_1_ = 1. Once *S*_config_ is characterised, we evaluate how energy is transferred through this system. The associated dissipation considering *E* as a current source is given by

(25) $$P_{diss_{I}}=I_{R1}^{2}R_{1}+I_{R2}^{2}R_{2}=I^{2}\frac{R_{1}R_{2}}{R_{1}+R_{2}}=I^{2}R_{eq},$$

where *R*_eq_ = *R*_1_*R*_2_/(*R*_1_ + *R*_2_). For a voltage source

(26) PdissV=V2Req

For both sources, the generated thermodynamic entropy rate is as follows:

(27) $${\dot{S}}_{therm}=\frac{P_{diss}}{T}$$

If we assume a constant temperature *T* and steady state, as in electronic circuit analysis, for a fixed time, we obtain $S_{therm}\propto P_{diss}$. We plot this in Figure 4. From now on, we use a normalized current source of 1 A, voltage source of 1 V, and integration time of 1 s in all future calculations (in steady state $S_{therm}={\dot{S}}_{therm}\cdot t$). We would like to point out that *S*_therm_ describes the conversion of electrical energy into thermal energy, whereas *S*_config_ describes the arrangement of the resistors and, thus, they are different.

**Figure 5:**
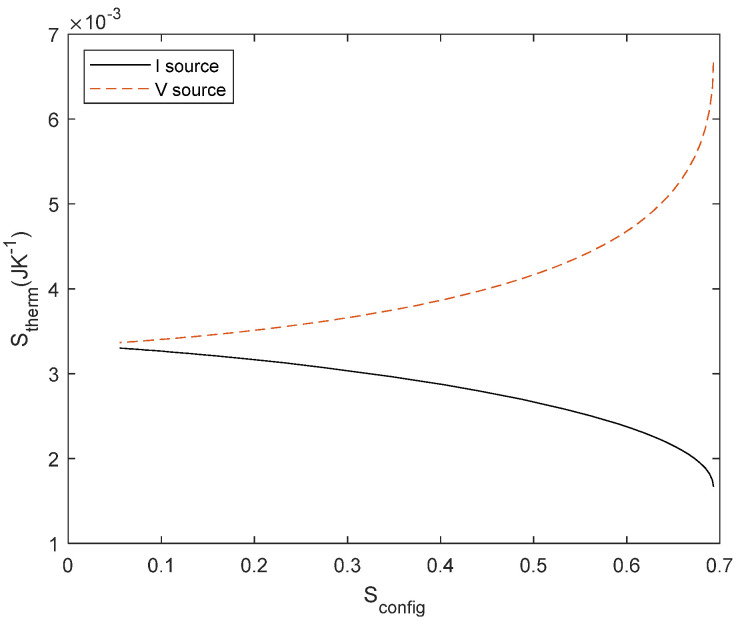
Thermodynamic entropy vs. Configurational entropy from a normalized current source (1 A, in black) and normalized voltage source (1 V, in dashed red line) and integrated for 1 s.

It is worth plotting the relationship between the two types of entropy for this circuit (illustrated in Figure 5) for a normalized *R*_1_ = 1 Ω by sweeping *R*_2_ for *R*_1_ ≤ *R*_2_ ≤ ∞. We consider *S*_config_ a dimensional and *S*_therm_ with units JK^−1^; using a common unit would scale one axis. Notice that the maximum *S*_config_ is the same as inferred from Equations (17) and (20). In the limit of *R*_2_→∞, according to Equations (25) and (26) *S*_therm_ converge to the same value. The interest of this result lies in the fact that *S*_therm_ increases with *S*_config_ for voltage but decreases for current, i.e., the gradient (*V*) dissipates more energy as the difference between resistors decreases, whereas the flow accommodates better as the difference between resistors increases. This relationship between the design of the circuit and the energy dissipation in the circuit seems to be useful in other fields: it could help to improve energy efficiency or adiabatic computing in electrical circuits or, in a more general approach, it might justify the constructal law [25,44]. We discuss these possibilities and further implications in Section 4.

Having introduced the relationship between configurational and thermal entropy, we can consider the case of two resistors at different temperatures, which was the main motivation for this study. The electrical properties of materials are temperature dependent. We consider standard resistors whose resistance depends on temperature as follows:

(28) $$R_{2T}\left(T\right)=R_{2}\left[1+\alpha \left(T_{2}-T_{1}\right)\right]$$

where *α* is the temperature coefficient of resistance and is valid for small temperature variations. The current divider becomes temperature-dependent at a steady state:

(29) $$I_{1}=I\frac{R_{2T}}{\left(R_{1}+R_{2T}\right)}$$

$$I_{2}=I\frac{R_{1}}{\left(R_{1}+R_{2T}\right)}$$

**Figure 6:**
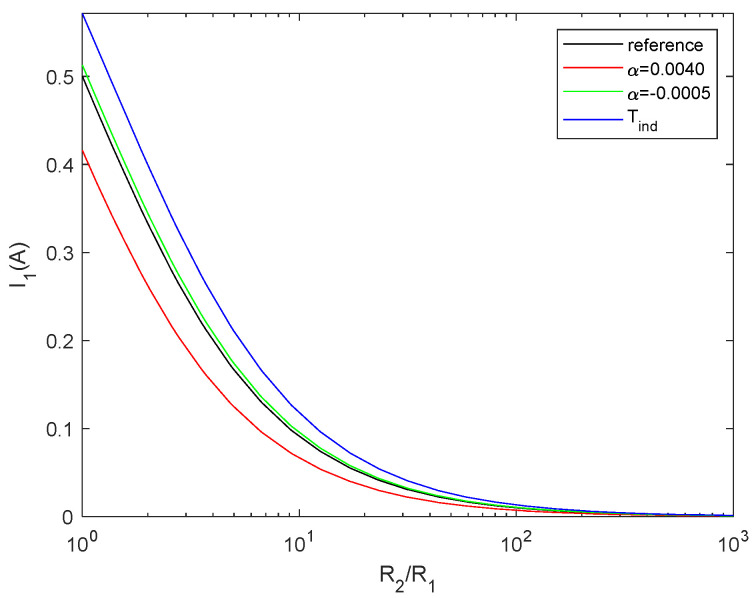
Current through *R*_2_ for reference case at constant temperature (black), temperature-dependent resistor with *α* = 0.0040 (red), temperature-dependent resistor with *α* = −0.0005 (green), and temperature-independent resistors (blue). All cases considered *T*_1_ = 300 K and *T*_2_ = 400 K. The difference between the blue curve and the reference curve indicates that it is not possible to modify the thermal dissipation without changing the structure of the material, i.e., the temperature coefficient term proportional to *α*.

The temperature coefficient can be either positive (for most metals) or negative (for germanium and carbon, for instance). In Figure 6, we plot the currents for the reference configuration (*α* = 0 and constant temperature), temperature-dependent resistors with *α* = 0.004 following Equation (20), temperature-dependent resistors with *α*= −0.0005 and temperature-independent resistors (described by Equation (14)). All examples are calculated with *T*_1_ = 300 K and *T*_2_ = 400 K. It can be seen that the curve for positive *α* is below the reference curve, as expected due to the increase in resistance with temperature, whereas for negative *α*, it is above the curve. With regard to the case described by Equation (14), we note that it is above the reference case, which is impossible, meaning that it is not possible to change the temperature of the material without changing its configurational properties. Therefore, in order to minimise the entropy generation, we have to consider the change in *S*_config_ due to thermal variation. This is a kind of Maxwell’s demon [45], where *α* = 0 means that we have a system (the resistors) that allows energy to be converted from electricity to heat without modifying the circuit, i.e., without assuming any change in the entropy of the system and violating the second law of thermodynamics.

**Figure 7:**
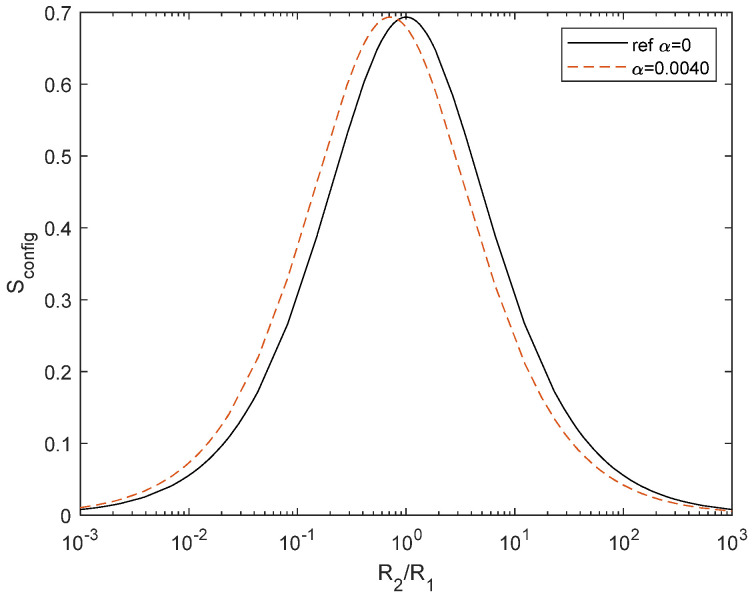
*S*_config_ change due to resistance variation on temperature with *α* = 0.0040 (dashed red line) with respect to the reference configuration (black).

As the resistance changes due to the temperature difference, *S*_config_ is modified accordingly:

(30) $$S_{config}=-\left[\frac{R_{2T}}{R_{1}+R_{2T}}ln\left(\frac{R_{2T}}{R_{1}+R_{2T}}\right)+\frac{R_{1}}{R_{1}+R_{2T}}ln\left(\frac{R_{1}}{R_{1}+R_{2T}}\right)\right]$$

the difference between the two cases is depicted in Figure 7. The overall magnitude does not change because we are plotting the normalized *R*_2_/*R*_1_, but the peak is shifted as follows:

(31) $$\frac{R_{2}}{R_{1}}=\frac{1}{1+\alpha (T_{2}-T_{1})}$$

**Figure 8:**
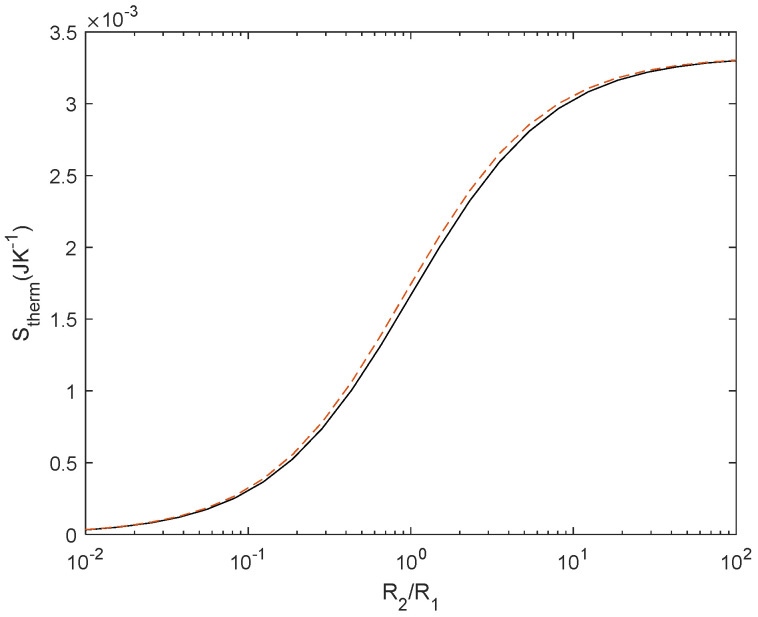
*S*_therm_ for reference case (black) and for temperature-dependent resistor with *α* = 0.0040 (in red) for a current source of 1 A.

Similar behaviour is found for *S*_therm_ (Figure 8), where we considered either a normalized current source of 1 A or a normalized voltage source of 1 V to estimate *S*_therm_.

**Figure 9:**
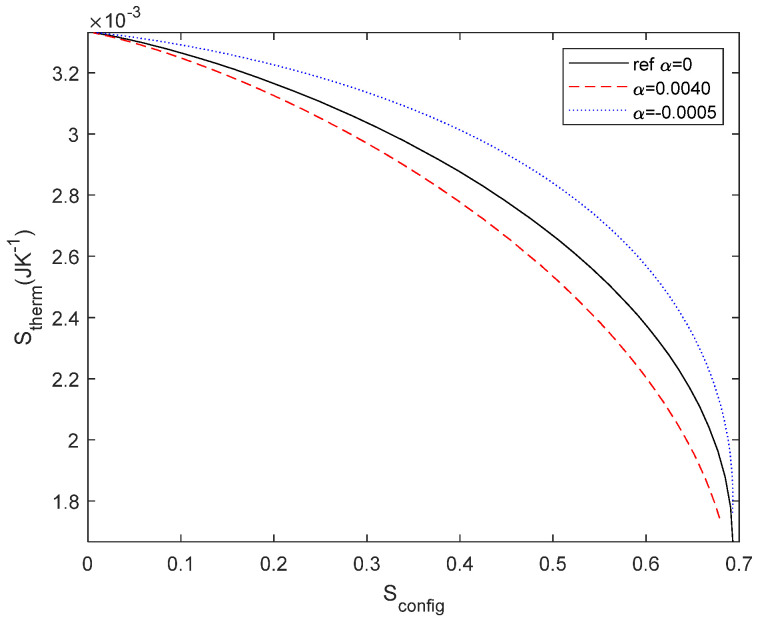
*S*_therm_ as a function of *S*_config_ for reference configuration without temperature variation (black), thermal-dependent resistor with *α* = 0.0040 (dashed red line) and resistor with *α* = −0.0005 (dotted blue line) for a current source of 1 A. The difference between curves is related to the configurational entropy change of the resistor due to the heat injection with the consequent temperature variation.

Finally, we evaluate *S*_therm_ with respect to *S*_config_ for the reference case and the temperature-dependent case (see Figure 9). The temperature difference modifies the internal resistance which implies that both *S*_config_ and *S*_therm_ are modified simultaneously. The difference between the curves is related to the entropy of the resistor, both configurational and thermal, due to the resistor’s structural changes associated with resistance variation and due to the different heat dissipations, respectively. As shown in Figure 6, it is not possible to modify only one type of entropy. If there is a change in the thermal dissipation, it must be due to a change in the material properties of the resistor. We also point out that this behaviour is the same for both positive and negative *α* and symmetrical for a voltage source, as in Figure 5.

### 3.2. Entropy in Series Resistors

We carry out the same procedures as for a parallel configuration in a series configuration (see circuit in Figure 2). To find ${\dot{S}}_{therm}$ for a voltage source, we can write the following:

(32) $${\dot{S}}_{therm}=\frac{P_{diss}}{T}=\frac{1}{T}\left[{\frac{1}{R_{1}}\left(\frac{R_{1}V}{R_{1}+R_{2}}\right)}^{2}+\frac{1}{R_{2}}{\left(\frac{R_{2}V}{R_{1}+R_{2}}\right)}^{2}\right]=\frac{1}{T}\frac{V^{2}}{R_{1}+R_{2}}$$

And for a current source, as follows:

(33) $${\dot{S}}_{therm}=\frac{P_{diss}}{T}=\frac{1}{T}I^{2}(R_{1}+R_{2})$$

**Figure 10:**
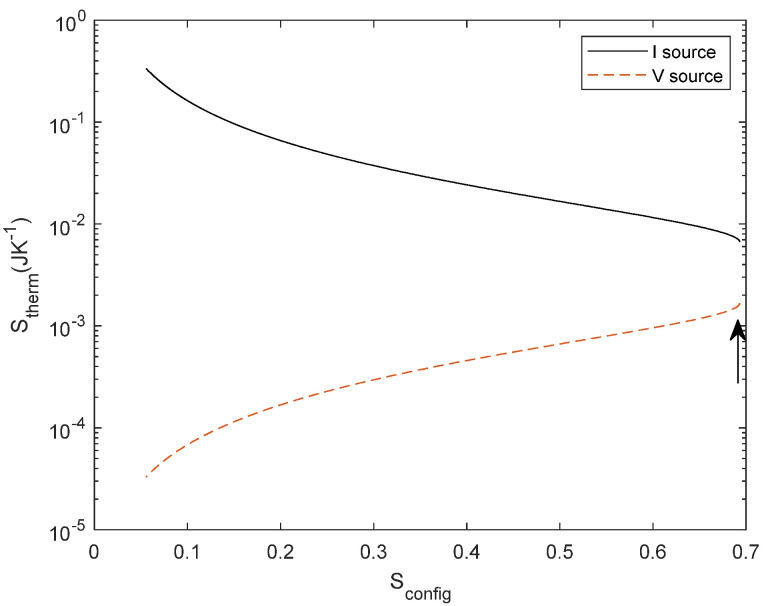
*S*_therm_ as a function of *S*_config_ for current source (black) and voltage source (dashed red line) for series resistors. The red point of maximum *S*_config_ and maximum *S*_therm_ corresponds to *R*_1_ = *R*_2_ as described by the maximum power transfer theorem and pointed out with the arrow.

The relationship between *S*_config_ and *S*_therm_ for series resistors is illustrated in Figure 10, as we have illustrated for parallel resistors in Figure 5. For a voltage source, we observe an increase of thermal dissipation with *S*_config_. The point of maximum *S*_config_ corresponds to *R*_1_ = *R*_2_. It is worth noting the relationship with the principle of maximum power transfer in a voltage divider, which occurs for *R*_1_ = *R*_2_. Maximum power transfer occurs when both types of entropy are at their maximum, i.e., when the difference between resistors is null and also the heat dissipation is at its maximum.

Now, consider the maximum power in the case of a current source. We can transform the circuit in Figure 9 using the Norton equivalent to obtain a current source of magnitude *I* = *V*/*R*_1_. In this case,

(34) $$P_{diss_{I}}=I^{2}\frac{R_{1}R_{2}}{R_{1}+R_{2}}={\left(\frac{V}{R_{1}}\right)}^{2}\frac{R_{1}R_{2}}{R_{1}+R_{2}}=V^{2}\frac{R_{2}}{R_{1}}\frac{1}{R_{1}+R_{2}}$$

which shows the validity of the maximum power transfer theorem for current sources. Note that Equations (34) and (25) are different and only equal in the case where *R*_1_ = *R*_2_, the limit case. Note, also, that this case is different from the case depicted in Figure 5, where the current is *I* and, here, it is *V*/*R*_1_.

**Figure 11:**
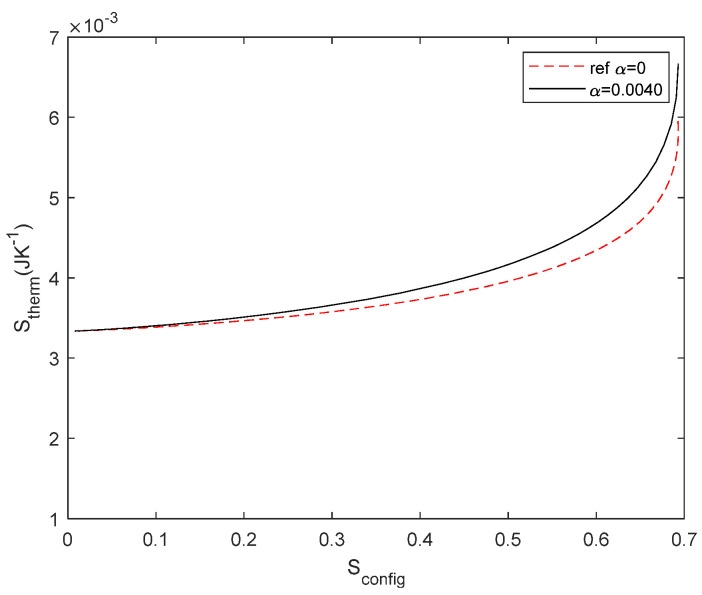
*S*_therm_ as a function of *S*_config_ for reference configuration (black) and thermal-dependent resistor (dashed red line) with *α* = 0.0040 for a voltage source. The difference between both curves is related to the entropy change of the resistor.

Finally, the temperature dependence for a series network, as illustrated in Figure 11, produces a change in the resistor according to Equation (31), the curve is slightly shifted similarly to the behaviour for a parallel network depicted in Figure 9.

### 3.3. Tree Shape Networks

**Figure 12:**
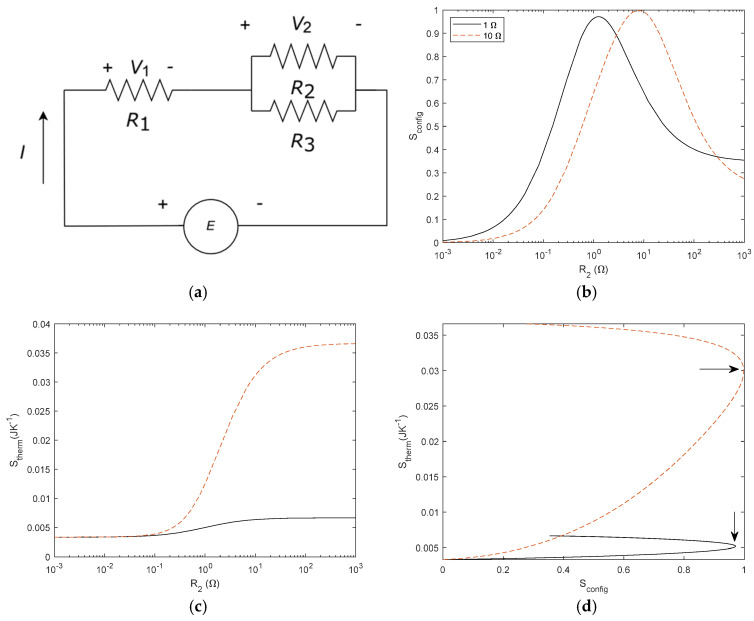
(**a**) Tree shape network with three elements. (**b**) *S*_config_ for 3 resistor circuit. *R*_1_= 1 Ω, *R*_2_ is the variable, *R*_3_ is studied for two cases: *R*_3_ = 1 Ω (black line), and *R*_3_ = 10 Ω (dashed red line). (**c**) S_therm_ for the circuit with *R*_3_ = 1 Ω (black line) and *R*_3_ = 10 Ω (dashed red line) *E* = 1 V and (**d**) S_config_ and S_therm_ relationship with *R*_3_ = 1 Ω (black line) and *R*_3_ = 10 Ω (dashed red line), *R*_2_ as a variable and *E* = 1 V. *R* symmetry is lost when *R*_3_ and *R*_1_ are different. The arrows point at the maximum *S*_config_.

**Figure 13:**
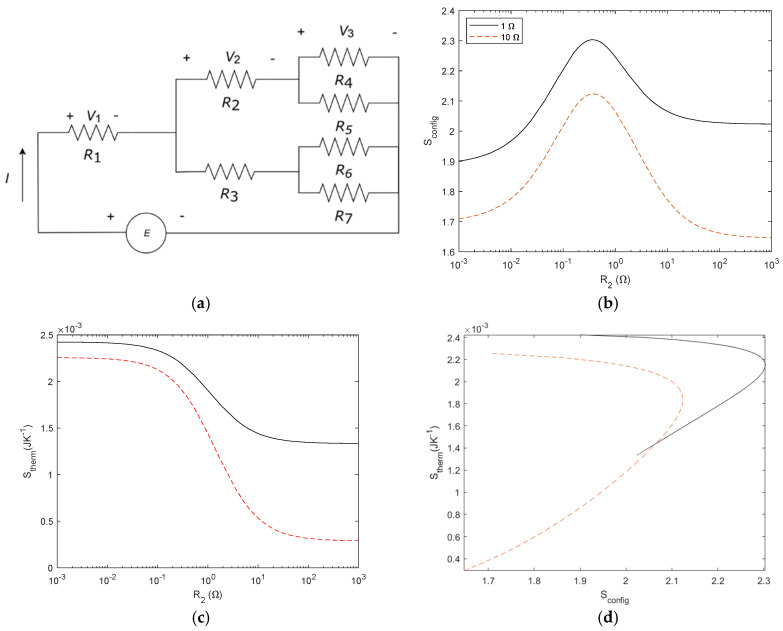
(**a**) Tree shape network with seven elements (**b**) *S*_config_ for 7 resistor circuit. *R*_2_ is variable and *R*_3_ = 1 Ω (black line) and *R*_3_ = 10 Ω (dashed red line). All other resistors are fixed to 1 Ω. (**c**) *S*_therm_ for the circuit with *R*_3_ = 1 Ω (black line) and *R*_3_ = 10 Ω (dashed red line) *E* = 1 V and (**d**) *S*_config_ and *S*_therm_ relationship with *R*_3_ = 1 Ω (black line) and *R*_3_ = 10 Ω (dashed red line), *R*_2_ as a variable and *E* = 1 V. Symmetry is lost when *R*_3_ and *R*_1_ are different.

Previous results, as shown in Figure 5, suggested the possibility of constructal law justification. To further investigate this possibility, we analyse typical structures proposed in the constructal law literature, such as river basins and capillary networks [25]. We consider two tree-shaped networks, with three and seven elements, respectively, and we compute *S*_config_ and *S*_therm_. The network structure and entropy results are given in Figure 12 for three elements and in Figure 13 for seven elements. We consider a voltage source of 1 V and compare *R*_3_ with 1 Ω and 10 Ω in both cases. The arrows shown in Figure 12d point to the maximum *S*_config_. The physical interpretation is given in the Section 4.

### 3.4. Circuits with More than One Source

**Figure 14:**
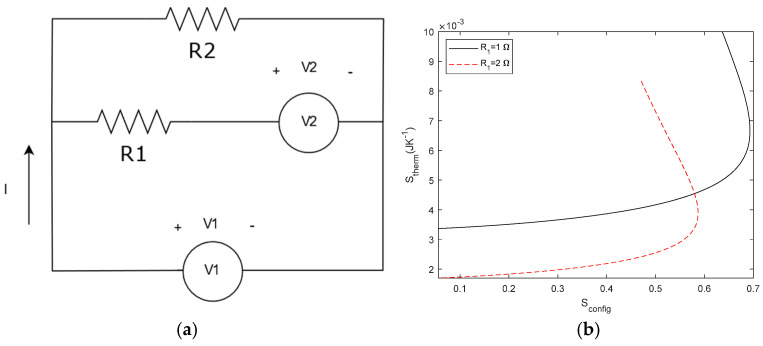
(**a**) Circuit with two voltage sources and two resistors. (**b**) S_config_ − S_therm_ relationship for *V*_1_ = 1 V, *V*_2_ = 2 V and *R*_1_ = 1 Ω or 10 Ω. *R*_2_ is the swept variable.

Once we have evaluated circuits with a different number of resistors, we now study circuits with more than one power source. We add a voltage source *V*_2_ in series to *R*_1_ with *E* as the voltage source (see Figure 14a). For *S*_therm_, we calculate the power dissipated in *R*_1_ and *R*_2_ by *V*_1_ and add the dissipated power by *V*_2_.

(35) $$P_{2}=\frac{V_{1}^{2}}{R_{2}}$$

(36) $$P_{1}=\frac{{\left(V_{1}-V_{2}\right)}^{2}}{R_{1}}$$

(37) $$P_{T}=P_{1}+P_{2}$$

The relevant question here is to analyse *S*_config_. When we studied the current and voltage dividers, *S*_config_ was given by the relationship between resistors. However, Shannon’s entropy relates the probability of the electrical to thermal dissipation in the circuit. Thus, it will depend on the different sources and resistors, so we consider the probability as the ratio of the power dissipated at each resistor with respect to the total power injected in the system. For the circuit depicted in Figure 14, substituting in Equation (16) with *p*(x) = *P*_i_/*P*_T_. we can write the following:

(38) $$S_{config}=-\sum_{i=1}^{n}p\left(x\right)\ln\left(p\left(x\right)\right)=-\left(\frac{P_{1}}{P_{T}}ln\frac{P_{1}}{P_{T}}+\frac{P_{2}}{P_{T}}ln\frac{P_{2}}{P_{T}}\right)$$

Which is a generalization of Equations (17) and (20).

### 3.5. Time Dependent Entropy in an R-C System

**Figure 15:**
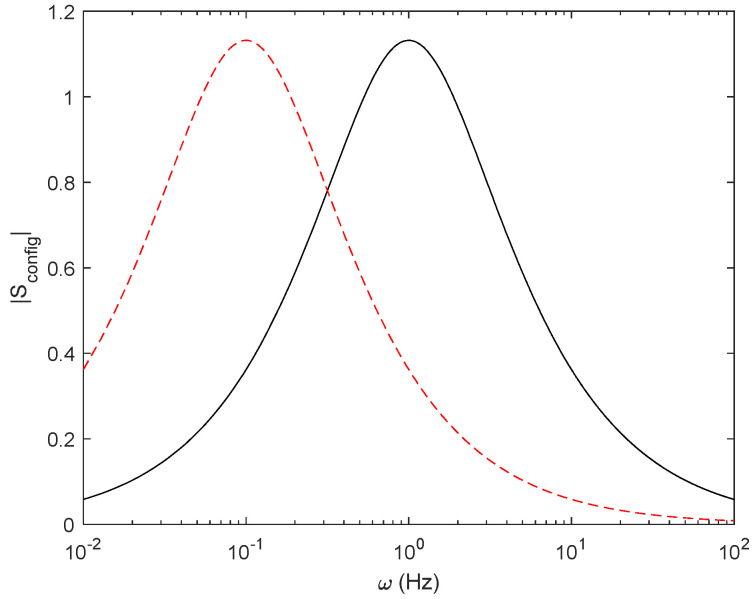
Modulus of S_config_ as a function of frequency for *C* = 1 F, *R* = 1 Ω (black), and *R*= 10 Ω (dashed red line).

**Figure 16:**
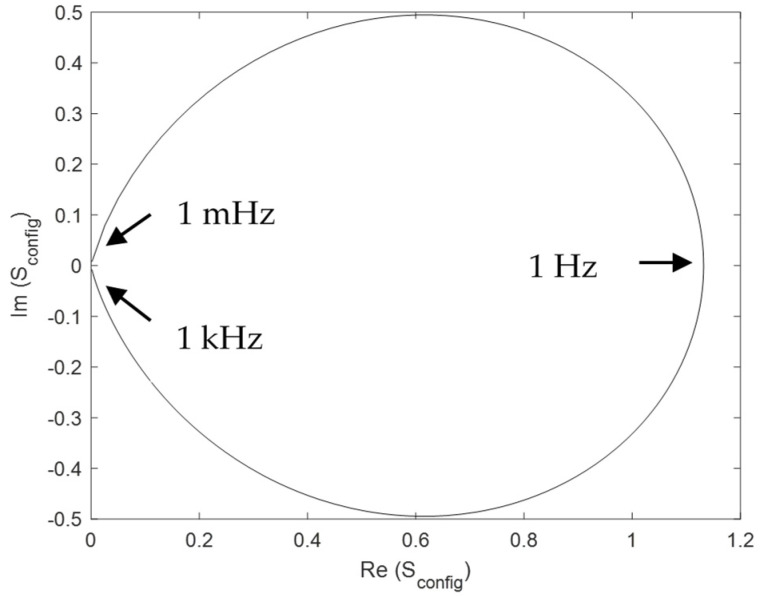
Nyquist plot for *S*_config_ for *R* = 1 Ω, *C* = 1 F, and 1 mHz < *ω* < 1 kHz.

We describe the results for a resistor in series with a capacitor (see the circuit in Figure 3). Following the same methodology described for resistors, we evaluate the configurational and thermal entropy. Thus, we briefly describe the behaviour of the entropy in terms of the degrees of freedom of the circuit, *R*, *C*, and *ω*, illustrated in Figure 15 as a function of frequency and as a Nyquist plot (Figure 16). We can see that the maximum configurational entropy is found at typical cut-off frequency ω=12π RC and is equal to 1.132. Also, in the Nyquist plot, we find that the maximum entropy corresponds to the only real point in the plot. Both results are obtained from Equation (21). Moreover, in the limit of low and high frequencies, *S*_config_ tends to be 0, which is reasonable since the capacitor is either short-circuited or open circuit and, thus, we have only one degree of freedom and entropy is null. It should be remembered that, at the cut-off frequency, the impedance modulus of the resistor and the capacitor are equal, which is consistent with the previous results of finding the maximum power transfer at the maximum configurational entropy.

**Figure 17:**
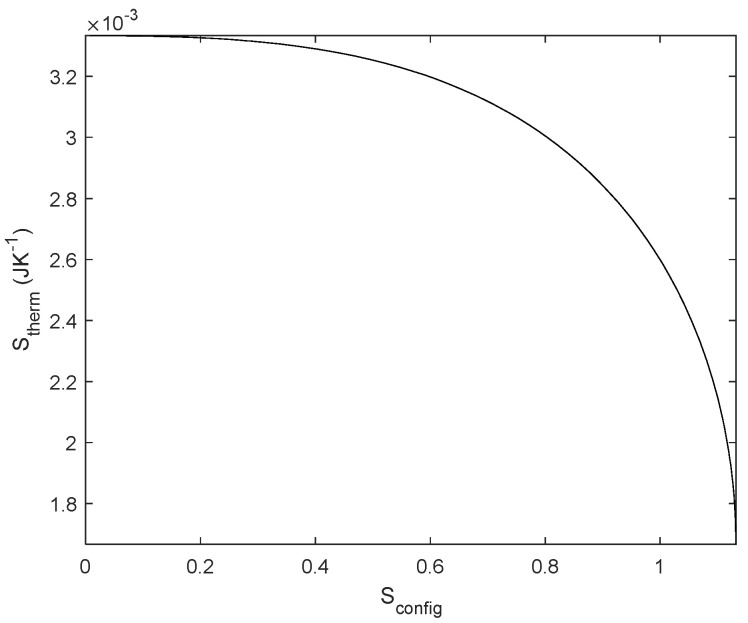
Relationship between S_config_ and S_therm_ for an *R-C* system (*R* = 1 Ω, *C* = 1 F, and *T* = 300 K). A similar behaviour to resistor circuits is found, showing the generality of the method for linear systems.

Once we have presented the configurational entropy, we analyse the relationship between circuit configuration and energy conversion (Figure 17). First, we consider *E* to be a constant voltage source. The energy stored in the capacitor when fully charged is Ec=12CV2. Integrating over the entire capacitor charge, the energy lost at the resistor during the charging process is ER=12CV2, which is independent of *R* and the frequency and, thus, *S*_config_ is null.

Secondly, we consider *E* as a sinusoidal source. In this case, the power dissipated at the resistor depends on the root mean square of voltage *V*_rms_, where *E* is the amplitude of the sinusoidal.

(39) $$V_{rms\_R}=\frac{R}{R+\left|Z_{c}\right|}\;\frac{\left|E\right|}{\sqrt{2}}$$

And the generated entropy is as follows:

(40) $${\dot{S}}_{therm}=\frac{{I_{rms}V}_{rms\_R}}{T}$$

The relationship between *S*_config_ and *S*_therm_ (integration of ${\dot{S}}_{therm}$ over time), as depicted in Figure 17, illustrates a similar behaviour to that found for resistors, justifying the feasibility of applying the method to linear systems in general.

### 3.6. Time-Dependent Entropy for Degradation

**Figure 18:**
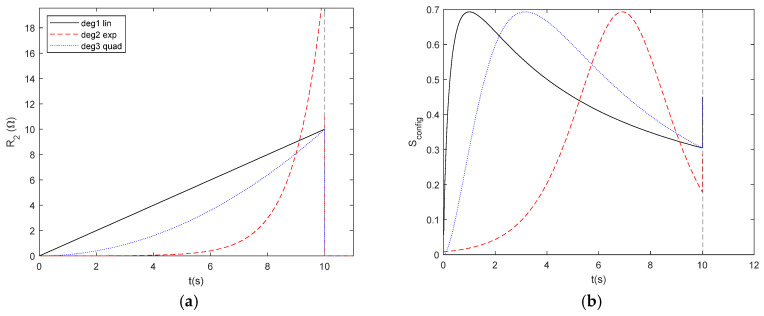

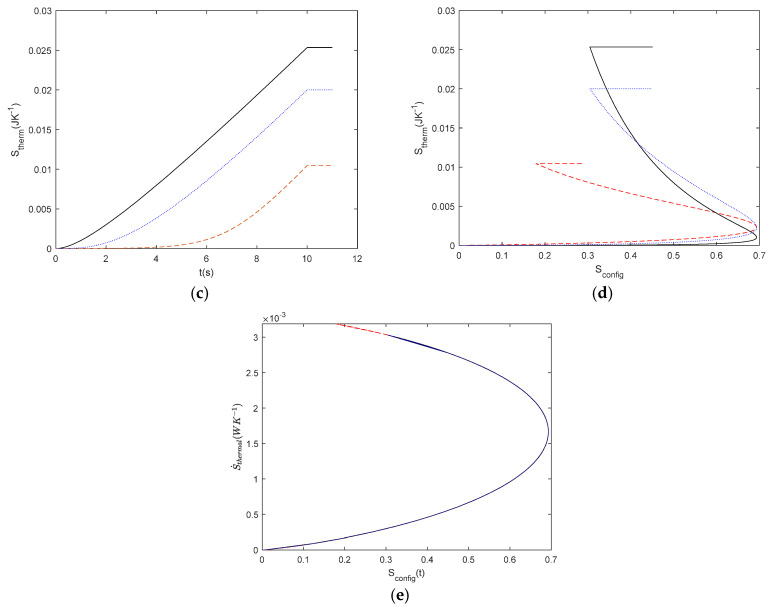
Time dependent profiles for degradation in a parallel *R*_1_//*R*_2_ circuit with *R*_1_ = 1 Ω, *T* = 300 K, and *I* = 1 A. (**a**) Time evolution of resistor degradation according to Equations (22)–(24). (**b**) Time dependent evolution of *S*_config_. (**c**) Time dependent evolution of *S*_therm_. (**d**) Relationship between *S*_config_ − *S*_therm_. (**e**) Relationship between *S*_config_ and $\dot{S}$_thermal_.

We consider the degradation functions proposed in the Section 2 to study the effect of the degradation of a resistor in a parallel resistor network (Figure 18a) as a function of time in order to obtain *S*_config_ (*t*) (Figure 18b), *S*_therm_ (*t*) (Figure 18c), and their relationship (Figure 18d). The abrupt change in behaviour at *t* = 10 s is due to the Heaviside function, representing the resistor breakdown. The different degradation mechanisms lead to different *S*_config_ − *S*_therm_ relationships, showing that the method is valuable for investigating systems that evolve with time. Moreover, it may be significant that the entropy generation rate $\dot{S}$_therm_ is independent of the degradation mechanism, as long as only *S*_config_ and $\dot{S}$_therm_ are involved in the degradation process due to *R*_2_, but it deserves further investigation.

## 4. Discussion

So far, we have obtained valuable results that are worth discussing: the entropy relation, the effect on constructal law and the theorem of maximum power transfer, and the effect of time on entropy in *R-C* circuits and degradation.

We can begin with entropy itself. In the literature, we found a discussion between Claude Shannon and John Von Neumann about entropy. C. Shannon said,

My greatest concern was what to call it. I thought of calling it ‘information’, but the word was overly used, so I decided to call it ‘uncertainty’. When I discussed it with John von Neumann, he had a better idea. Von Neumann told me, ‘You should call it entropy, for two reasons. In the first place your uncertainty function has been used in statistical mechanics under that name, so it already has a name. In the second place, and more important, no one really knows what entropy really is, so in a debate you will always have the advantage’.

Whether the story is true or not, it is certain that the concept of entropy still generates some debates on its interpretation and, as a physical magnitude and a mathematical property, it is worth devoting more research effort to it. We use the quotation to point out that they were probably both right. Entropy is a mathematical function that can be applied in different disciplines, but it has a different physical meaning in each problem. As mentioned above, Jaynes prevents us from confusing information entropy and thermodynamic entropy unless we are dealing with thermodynamic problems. So, what we have tried to investigate in this paper is not whether they are the same thing, but how they are related, as there are two types of entropy describing different characteristics of the system: this is the purpose of relating *S*_config_ to *S*_therm_. This correlation is possible because it is a consequence of Ohm’s law, that relates a flow, i.e., the current, and a gradient, i.e., the voltage with the entropy generation. This type of relationships is the basis of irreversible thermodynamics and, from this approach, the second law is introduced in the circuit analysis. Once we have introduced entropy, the introduction of the probability approach to the circuit description is easier to interpret. Thus, Kirchoff’s laws guarantees charge and energy conservation, whereas Ohm’s law allows for the introduction of the second law. Hence, *S*_therm_ is clearly related to energy conversion, i.e., when the energy enters the circuit, it is converted into heat. *S*_config_ is related to network structure, and is either decided by us, i.e., we decide whether to design a series or parallel circuit, or it evolves naturally over time due to degradation induced by energy dissipation. It is important to note that, if the relationship *R*_2_/*R*_1_ ratio changes due to energy dissipation, we have a causal relationship between *S*_config_ and *S*_therm_, as illustrated in Figure 5. Conversely, if we change the ratio arbitrarily or manually, there is a correlation, but not a causal relationship between *S*_config_ and *S*_therm_. The same happens with temperature, and here we find the origin of the problem inferred from Equation (14). We find that the change in entropy of the resistor (system) was not previously considered when its temperature changed. Taking this into account, the relationship *S*_config_ − *S*_therm_ clarifies the fact that entropy is a function of state of *S*_config_ and *S*_therm_ for the network system, but it is also needed to consider the entropy change in the universe to introduce the change in the network.

**Figure 19:**
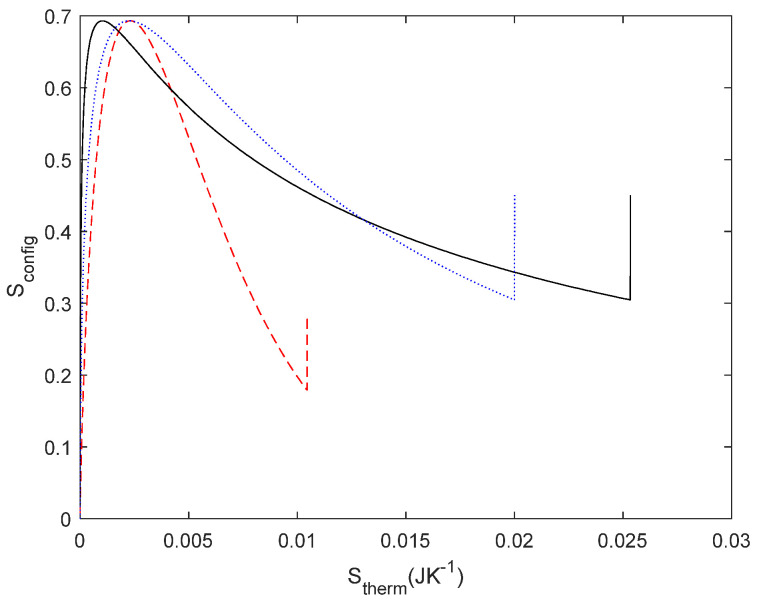
Relationship between *S*_therm_ − *S*_config._ It is the same data from Figure 18d with the axis exchanged.

In order to investigate both types of entropy, as to our knowledge, the entropy of a system is not usually correlated in complex systems; thus, we investigated how energy is transformed in a linear system as a function of the network configuration. On the one hand, *S*_config_ was derived from a probability of the electrical circuit; on the other hand, *S*_therm_ is inferred from thermodynamic relations. A consequence of deriving entropy from a probability is that we can deal with causal and not-causal systems. Obviously, *S*_therm_ is causal because it is governed by the laws of thermodynamics. But *S*_config_ is not causal, the network change is decided by the design of the electric circuit. In probability, if the system is not causal, the probability spaces are likely to be independent in many cases and, thus, the probabilities can be computed as independent. In our opinion, this approach allows an interdisciplinary approach to complex problems, as it allows us to deal with different properties of the system with the same magnitude. For instance, the studied *S*_config_ and *S*_therm_ are independent as long as *S*_config_ is not modified by the same energy. This explanation is consistent with the constructal law, as we proposed in the Section 3, which states that “for a finite size flow to persist in time (to survive) it must evolve in such a way that it provides easier and easier access to the currents that flow through it” [25]. Several examples of internal organization have been proposed, such as river basins, city streets, and railways maps [25]. To our knowledge, it has not been illustrated using this approach, i.e., the correlation between a geometrical entropy and a flow entropy, which can help to clarify its understanding. For a better understanding of this, we considered the typical examples of this law, such as tree-shaped structures that can be found either in rivers or blood capillaries. To gain a deeper insight into this result, we obtained the entropy correlation for tree-shaped structures with three and seven elements (see Figure 12 for three elements and Figure 13 for seven elements). In these networks, the symmetry of the resistor configuration is broken and, thus, *S*_config_ (*R*_2_/*R*_1_) is no longer symmetric. It is important to note that the maximum of *S*_config_ corresponds to an extreme of the *S*_therm_. As indicated by Bejan [25], the design of the structure organizes to fit the flows. From our results, and in other words, the organisation obeys a causal relationship between the energy involved in the process and the final network structure. From Bejan’s definition, we had understood that design configured the energy *S*_config_ − *S*_therm_ but it seems to be more convenient to plot *S*_therm_ − *S*_config_. For instance, replotting Figure 18d with the axis exchanged, it is easy to write a function *S*_config_ = *f*(*S*_therm_) as illustrated in Figure 19 and find the maximum as follows:

(41) $$\frac{dS_{config}}{dS_{thermal}}=0$$

The correlation between both types of entropy leads to a maximum in which the constructal law is satisfied. This approach therefore opens up the possibility of exploring a law for the dual (gradients), which has not been considered before, since gradients behave similarly to flows, as well as exploring causal and non-causal interdisciplinary problems.

Continuing with the constructal law applied to resistors, we can also interpret that Equation (17) depends on geometry, if we consider that the resistance is given by *R* = *ρ l*/*A*, where *ρ* is the resistivity, *l* is the length, and *A* is the area of the resistor. This agrees with the relationship between geometry design and energy dissipation given in the law.

Another interesting result concerns the theorem of maximum power transfer. This theorem is traditionally derived from a minimisation process of the energy transferred. In Figure 10, we found that the minimum point corresponds to the theorem. It is not surprising that this theorem depends simultaneously on the network and thermal entropy, since its classical derivation minimises the power of the network. However, using our approach *S*_config_ = *f*(*S*_therm_), we can have a better understanding of the power transfer not only at the extremal point but as a function of the network configuration, allowing further predictions of circuit performance.

Regarding the increase in information with more resistors or power sources, we can point out that changing the power sources affects the energy conversion but not the network configuration and, thus, while *S*_therm_ changes, *S*_config_ remains the same. On the contrary, increasing the number of resistors in the network introduces an entropy term for each resistor, thus changing *S*_config._ This is an interesting issue that we can discuss using the three-resistors circuit illustrated in Figure 12a. From an electrical point of view, we can connect the resistors in parallel and in series to find the equivalent resistor. The dissipated power will be the same for *R*_1_, *R*_2_, and *R*_3_, for *R*_1_ + *R*_2_//*R*_3_, or for *R*_eq_ = (*R*_1_ + *R*_2_//*R*_3_), where//means connected in parallel. However, the entropy associated with each case, assuming all resistors with *R* = 1 Ω, are 0.9634, 0.6365, and 0, respectively. Obviously, the entropy decreases as the circuit simplifies.

Time-dependent results are also interesting. On the one hand, there is the degradation studies; on the other hand, there is the complex entropy in *R-C* systems. Degradation is a causal constraint of network modification due to energy dissipation, which can be described by *S*_therm_. We proposed three arbitrary time-dependent curves. Previously, we had already investigated the physical degradation of resistors [34] and capacitors [35] in terms of ${\dot{S}}_{thermal}$. In degradation analysis, it is usually stated that the breakdown takes place when an entropy threshold is reached; that is, the system cannot hold more thermal entropy [39]. In fact, during our degradation experiments in [34], we pointed out that the thermal entropy threshold could explain either fatigue wear or breakdown in the resistor. However, as these two degradation mechanisms modify the internal structure of the material, it seems reasonable to investigate them in terms of *S*_config_. Results in Section 3.6 illustrate this approach, i.e., the material degradation leads to a resistance variation which leads either to a change in *S*_config_ or *S*_therm_, as illustrated in Figure 18. An interesting result, which could be worthy of further research, is that the relationship between *S*_config_ and ${\dot{S}}_{therm}$ is invariant to the degradation mechanism, as long as they are the only types of entropy involved in the process. However, *S*_config_ and *S*_therm_ are dependent on the degradation mechanism. Both results can be useful for degradation characterisation in order to complement the approaches given in [34,39].

The presence of imaginary entropy in an *R-C* configuration is related to the time-dependent behaviour of the capacitance. However, it is striking that, when the real part is maximum, and the imaginary part is zero, the entropy is maximum, which also corresponds to the same impedance modulus of the resistance and the capacitance at the point of maximum power transfer ratio. This behaviour could be further investigated in terms of active and reactive power.

To conclude this section, after discussing the theoretical implications of entropy correlations, we suggest possible practical applications that could benefit from these results. First, electrical circuit analysis can benefit from an additional criterion to improve energy efficiency, which can be of interest in the field of adiabatic computing. Moreover, for our interest, it may be useful in microgrid optimization or in battery performance analyses, which are usually described using lumped models in non-equilibrium conditions. The introduction of both types of entropy may help to better understand battery performance when degradation processes take place. Finally, we have correlated *S*_config_ and *S*_therm_ in electrical circuits, which are linear systems. We expect that this analysis can be useful in other linear systems, either in mechanics or in fluid applications.

## 5. Conclusions

In conclusion, the thermodynamic analysis of a simple electric circuit has yielded valuable conclusions, as we have clarified relationships between types of entropy. Firstly, we have related two different types of entropy that describe two different characteristics of the system. In particular, the relationship between configurational entropy and thermal entropy in different linear electric circuits provides a clear illustration of how energy transformation is related to the structure of the system upon which it is transformed. Secondly, the relationship between current flow and geometric design is highlighted as an explanation for the constructal law, which describes how a system must change to accommodate an entropic change. Thirdly, the change in configurational entropy due to a temperature difference is demonstrated to justify the thermal dissipation of the electric circuit. Fourthly, it is shown that the maximum power transfer theorem satisfies an entropy maximum, both for thermal and configurational entropy. Finally, the methodology was extended to *R-C* systems, resulting in a dependence similar to that observed in resistors. It is anticipated that these results, derived from simple examples, can be generalized to more complex systems.

## Data Availability

Dataset available on request from the authors.
